# Effects of Open- and Closed-Label Nocebo and Placebo Suggestions on Itch and Itch Expectations

**DOI:** 10.3389/fpsyt.2019.00436

**Published:** 2019-06-21

**Authors:** Stefanie H. Meeuwis, Henriët van Middendorp, Antoinette I.M. van Laarhoven, Dieuwke S. Veldhuijzen, Adriana P.M. Lavrijsen, Andrea W.M. Evers

**Affiliations:** ^1^Health, Medical and Neuropsychology Unit, Faculty of Social and Behavioural Sciences, Institute of Psychology, Leiden University, Leiden, Netherlands; ^2^Leiden Institute for Brain and Cognition, Leiden University Medical Center, Leiden, Netherlands; ^3^Department of Psychiatry, Leiden University Medical Center, Leiden, Netherlands; ^4^Department of Dermatology, Leiden University Medical Center, Leiden, Netherlands

**Keywords:** placebo, nocebo, itch, suggestion, pruritus

## Abstract

Placebo and nocebo effects have been shown to influence subjective symptoms such as itch. These effects can be induced by influencing outcome expectations through, for example, combining the application of an inert substance (e.g., a cream) with verbal suggestions on the anticipated effects of this substance. Interestingly, placebo effects also occur when it is known that a treatment is inert (i.e., open-label placebo). However, no study to date has examined the efficacy of negative and positive verbal suggestions under similar open-label and closed-label (i.e., concealed placebo/nocebo) conditions in itch. A randomized controlled between-subjects study design was applied in which healthy volunteers (*n* = 92) were randomized to 1) an open-label positive verbal suggestion group, 2) a closed-label positive verbal suggestion group, 3) an open-label negative verbal suggestion group, or 4) a closed-label negative verbal suggestion group. Verbal suggestions were made regarding the topical application of an inert substance. Itch was evoked experimentally by histamine iontophoresis at baseline and again following suggestions. Itch expectations, self-reported itch during and following iontophoresis, and skin response parameters were measured. Positive suggestions were found to result in significantly lower expected itch than were negative suggestions in both open- and closed-label conditions. No effects of the suggestions on itch during iontophoresis were found, but significantly lower itch was reported in the 4 min following iontophoresis in the (combined open- and closed-label) positive compared with negative verbal suggestion groups. In addition, a smaller increase in skin temperature was found in the positive compared with negative suggestion groups. The findings illustrate a potential role of (open- and closed-label) placebo for optimizing expectations and treatment effects for itch in clinical practice.

**Clinical Trial Registration:** Netherlands Trial Register, trial number: NTR6530.

## Introduction

Itch is the most common somatosensory symptom in dermatological conditions. It is a hallmark symptom of atopic eczema (1), and its prevalence in psoriasis is high (2). Moreover, itch is a common symptom of various other disorders, including kidney failure, liver disease, cancer, allergy, and diabetes mellitus (3–5). Due to its high prevalence—approximately 8% of the general population and over 50% of dermatological patients—the burden of itch and its impact on society are high (6, 7). Often, patients report significantly lowered quality of life, increased depressive and anxious symptoms, and sleep disturbances due to chronic itch (8). While current treatments aim to suppress itch through pharmacological interventions, oftentimes, limited effects and significant side effects are reported (3, 9). As such, it is important to identify factors that contribute to treatment efficacy.

One of the factors that may be especially relevant for treatment outcomes is the placebo effect. Placebo effects are defined as beneficial effects of otherwise pharmacologically inert substances (10, 11) and have been studied in a variety of medical conditions and symptoms, including itch and pain (12–14). Multiple pathways through which these effects can be elicited have been identified, including associative learning processes, social learning, or instructional learning (12, 15–17). Within these pathways, expectancy is a key component. To illustrate, a positive expectation may be elicited through past experiences with the beneficial effects of a certain type of medication (associative learning), through observation of treatment efficacy in others (social learning), or through receiving positive (verbal) information about a treatment (instructional learning) (17). In turn, this positive expectation can then result in psychoneurobiological changes and symptom reduction (18, 19). On the other hand, when expectations regarding a treatment outcome are low or negative outcomes are expected, symptoms may worsen or the occurrence of treatment side effects may increase, known as the nocebo effect (12, 20).

The current literature indicates that at least 30% of itch reduction in clinical practice might be attributable to placebo effects (21). Placebo and nocebo effects can be experimentally induced for itch by changing expectations through verbal suggestions regarding inert treatments or through the use of associative learning mechanisms (22–28). However, not all studies confirm these findings (28, 29). In addition, there is some evidence that it may be necessary to combine multiple placebo induction methods (e.g., associative learning and positive suggestions) and that a single induction method may not be sufficient to elicit significant placebo effects (22). Hence, further study of the circumstances under which placebo effects may be elicited for itch is relevant.

Most studies on placebo effects take on a traditional approach, in which patients or healthy individuals are told that a pharmacologically effective substance (e.g., a pill or cream) is given, whereas in reality, the substance is pharmacologically inert (30, 31). While this concealing or deceptive approach is useful for studying the underlying mechanisms of placebo effects in general, it may become problematic when it comes to utilizing these effects in clinical practice, where concealment or deception regarding the treatment provided brings along ethical issues. For a long time, it was believed that this would prevent strategic use of the placebo effect in clinical practice (30). In the past years, however, studies have demonstrated that placebo effects can also occur when it is explicitly told that, although a given substance is inert, placebo effects may still help in alleviating symptoms. These so-called open-label placebo effects have been found to significantly reduce symptoms of irritable bowel syndrome, depression, attention deficit hyperactivity disorder, chronic low back pain, and allergic rhinitis (31–39). Most of these studies induce open-label placebo effects through a combination of an attribute (e.g., an inert pill) that may trigger previously learned associations between medicine and symptom reduction in general, and a scripted briefing in which the positive effects of placebo pills are emphasized (a suggestive framework) (31–34, 36–38). Findings on whether these effects can be attributed to the provided pill or the provided explanation alone are contradictory (35, 39, 40).

In view of the previous findings, further research is needed to demonstrate the efficacy of both open-label and closed-label (i.e., concealed) placebo effects for itch. It is not yet known whether effect sizes of open-label and closed-label placebo effects are comparable. Moreover, no study to date has investigated whether nocebo effects can be induced under both closed-label and open-label conditions for itch. To this end, we investigated in the current study whether negative or positive outcome expectations, induced by a suggestive framework (negative and positive verbal suggestions, provided in an open-label and closed-label context) combined with an attribute (an inert tonic), can influence self-reported itch during an experimental itch induction by histamine in healthy volunteers. We primarily tested the effects of the positive and negative suggestions on itch by combining open- and closed-label groups. Secondarily, we tested these effects for open-label and closed-label contexts separately to see whether these effects were comparable, and we investigated the effects of suggestions on other markers of the response to this test, for example, the physical skin response to histamine. We expected a decrease in itch following positive verbal suggestions compared with an increase in itch following negative verbal suggestions for both the open-label and closed-label conditions.

## Materials and Methods

The study was approved by the Medical Ethical Committee at the Leiden University Medical Center, the Netherlands (NL58792.058.16), and registered in the Dutch Trial Register (NTR6530). The study was performed in accordance with the Declaration of Helsinki (41). All participants provided written informed consent.

### Participants

Healthy male and female participants were recruited through advertisements at Leiden University and through social media (e.g., Facebook). Inclusion criteria consisted of an age between 18 and 35 years and a good understanding of the Dutch written and spoken language. Interested volunteers were excluded in case of self-reported severe somatic or psychological morbidity that could interfere with the participant’s safety or study protocol [e.g., heart or lung diseases, histamine intolerance, or Diagnostic and Statistical Manual of Mental Disorders - Fourth Edition Text Revised (DSM-IV-TR) psychiatric diagnoses]; current chronic itch or pain complaints; current use of analgesics, anti-inflammatory medication, antihistamines, or antibiotics; and (suspected) pregnancy. Participants were asked to refrain from the consumption of heavy meals, caffeine, and smoking 2 h, exercise 12 h, and alcohol as well as drugs 24 h prior to the sessions to prevent potential influences on study outcomes. Adherence to these lifestyle guidelines, as well as the exclusion criteria, was verified at the start of each session by means of interviewing.

### Study Design

**Figure 1:**
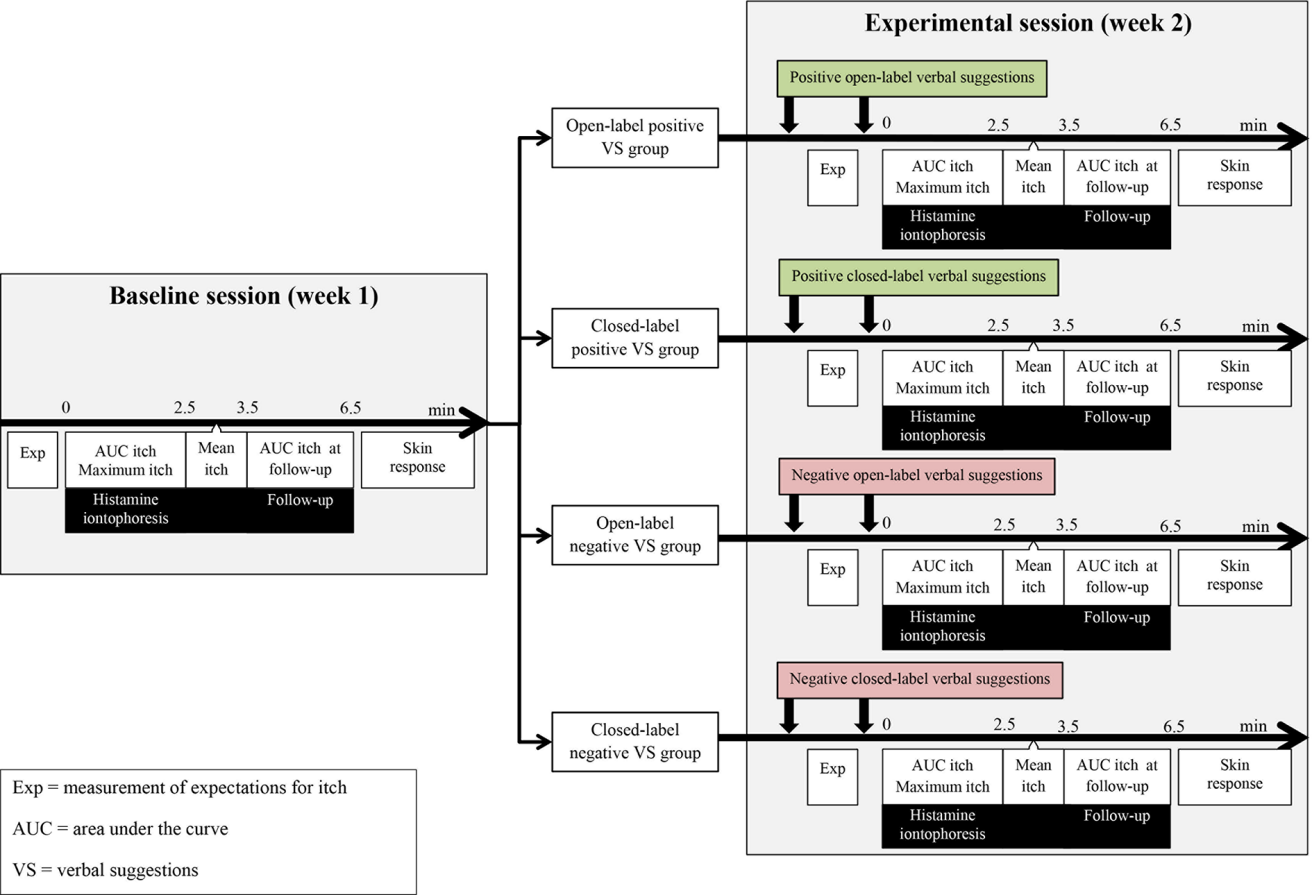
Overview of the design of the study and the measurement schedule for the different verbal suggestions (VS) groups.

A between-subjects, single-blinded, randomized controlled experimental trial design was applied. Eligible participants were randomized to (I) an open-label positive verbal suggestion (VS) group, (II) a closed-label positive VS group, (III) an open-label negative VS group, or (IV) a closed-label negative VS group. Randomization sequence was acquired using an online random number generator (www.random.org, Dublin, Ireland). Allocation was not concealed for experimenters. Participants were invited for a baseline and an experimental session, which were timed 1 week apart. An overview of the study design and measurement schedule is provided in **Figure 1**.

### Measures and Materials

#### Verbal Suggestions

Before the study, participants were informed that the study aimed to investigate individual differences in the sensitivity to itch and the role of psychological factors in explaining these differences. They were informed that the itch induction method would elicit a response similar to a mosquito bite (e.g., that their skin may become red and swollen). During the experimental session, participants were told that, prior to itch induction, a tonic would be applied that influences sensitivity to itch. In reality, this tonic was a pink-colored skin disinfectant (Orphi Pharma B.V., Dordrecht, the Netherlands). The itch induction during the baseline session was used as a reference point for the suggestions. In the positive VS groups (I and II), the following suggestion was given: “This tonic has an itch-reducing effect and will make the skin less sensitive to itch. From previous research we know that the application of this tonic will reduce itch for most people, meaning around 95 percent of the studied people. As such, we expect that you will experience less itch, compared to the previous test.” Participants in the negative VS groups (III and IV) were given the same information, but negative words were used instead of positive words (e.g., “itch-increasing” rather than “itch-reducing”).

When participants were allocated to one of the two open-label groups, additional instructions were given. For the positive VS group, these were: “I just told you that the tonic reduces itch. In fact, the tonic is a placebo. From research we know that the expectation that a remedy reduces itch will really cause people to experience less itch. This is caused by different processes, for example itch-reducing substances that are released in the brain. These substances are also released when people know that they receive a placebo. So, even though I told you this, you will likely experience less itch during the test.” For the open-label negative VS groups, negative words were again used in the instructions instead of positive words. During application of the tonic, the provided suggestions and, if applicable, open-label instructions were briefly repeated in a single sentence.

#### Expected Itch

Expected itch in response to histamine iontophoresis was assessed on a Numeric Rating Scale (NRS) ranging from 0 (“no itch”) to 10 (“worst itch imaginable”). Participants rated the amount of itch they expected to experience during iontophoresis twice: once at the start of the baseline session and once during the experimental session, following the verbal suggestions but prior to histamine iontophoresis.

#### Histamine Iontophoresis

Histamine was applied to the skin by transdermal iontophoresis (Chattanooga Group, Hixson, TN, USA). This method has been previously validated and reliably induces itch in healthy populations (22, 28, 29, 35). A 0.6% diphosphate (equivalent to 1% histamine dihydrochloride) histamine solution was prepared in distilled water with propylene glycol and hypromellose 4,000 mPa by the local pharmacy. In preparation of iontophoresis, the skin was cleaned with either a transparent disinfectant (alcohol 70%; baseline session) or a pink-colored disinfectant (0.5% chlorhexidine in alcohol 70%, with rhodamine; experimental session) suggested to be itch-reducing or itch-increasing, depending on placebo or nocebo condition. A 2.5-cc electrode (Iogel, Iomed, DJO Global, Hannover, Germany; active surface: 11.7 cm^2^) was treated with the histamine solution and applied to the volar side of the non-dominant forearm. A reference electrode was placed on the volar side of the upper arm. The electrode nodes were spaced approximately 10 cm apart. Histamine was applied to the skin by iontophoresis with a current level set at 0.4 mA for 2.5 min, following which the electrodes and any residual histamine were removed from the skin.

#### Self-Reported Itch

Self-reported itch in response to histamine iontophoresis was assessed using the same 0–10 NRS as described under Expected Itch. During iontophoresis, participants continuously rated itch using a vertical bar slide depicting the NRS. Scores were sampled at a 10-Hz rate using E-Prime 2.0 (42). Directly following iontophoresis, mean itch was verbally assessed by asking participants how much itch (on a 0–10 scale) they experienced in general during the test. From 1 to 4 min after iontophoresis, participants were asked to rate self-reported itch every 30 s on the bar slide as a follow-up period. The primary study outcome was the area under the curve (AUC) of itch during the 2.5 min of iontophoresis. Secondary outcomes were maximum itch reported during the 2.5 min of iontophoresis, verbally assessed mean itch, and AUC itch during the 4-min follow-up. AUC of itch and maximum itch during iontophoresis were computed using MATLAB Release 2012b (The MathWorks, Inc., Natick, MA, USA).

#### Subjective Skin Response

Participants filled in the Sensitive Scale-10 (SS-10) (43) to measure their subjective skin response. The SS-10 contains 10 items, of which 9 items assessed specific skin symptoms (e.g., itch, pain, general discomfort, and heat sensations). Participants were asked to rate in what intensity they had experienced these symptoms over the past 3 days as a baseline measurement, as well as during histamine iontophoresis. Symptoms were rated on NRS ranging from 0 (“zero intensity”) to 10 (“intolerable intensity”). An additional symptom (“redness of the skin”) was assessed on a 0–10 NRS (43). Total scores were calculated by summing all items and ranged from 0 to 100. Cronbach’s alpha ranged from .83 to .87 in the current study for baseline and post-iontophoresis assessments of the SS-10.

#### Physical Skin Response

Wheal size and flare areas following histamine application were measured after the 4-min follow-up period after the iontophoresis test. The size of the skin response was measured by drawing the outline of the redness and thickening of the skin onto a 1-cm^2^ gridded transparent sheet with a 0.4-mm black permanent marker (Staedtler, Germany). The sheets were scanned and then retraced using ImageJ software (44), after which the wheal and flare area (in cm^2^) were calculated. In addition, skin temperature was measured following iontophoresis, using a handheld infrared digital thermometer (accuracy ± 2.0 °C, resolution 0.1 °C; BaseTech, Conrad Electronic Benelux B.V.). Measurements were taken with the thermometer held vertically and approximately 1 cm above the center of the histamine application area. To control for individual differences in skin temperature, a baseline measurement was taken prior to iontophoresis, with change from baseline temperature being used as the outcome measure.

### Procedures

Prior to participation, written information regarding the study was provided, and volunteers were asked to fill in an online questionnaire assessing the study’s inclusion and exclusion criteria. When volunteers were considered eligible for participation, they were invited to the lab for a 30-min baseline session and a 45-min experimental session, which were timed 1 week apart. At the start of the baseline session, the study procedures were explained, and written informed consent was provided. Next, personality questionnaires were administered, which are not further described here as they are unrelated to the aim of the current study. Baseline measurements of itch expectation and subjective skin responses in the past 3 days were taken. Next, the skin of the non-dominant forearm was disinfected, and electrodes were placed on the arm, after which the histamine test was conducted. Measurements of itch and physical skin responses were taken, followed by an assessment of subjective skin responses. After 1 week, the experimental session took place. First, the general procedure of the second session was explained, and verbal suggestions were given (the content of which depended on group allocation). Measurements of post-VS expected itch and of subjective skin responses in the past 3 days were taken. Next, the skin was cleaned using the pink disinfectant, during which the verbal suggestions were briefly repeated. Histamine iontophoresis was conducted; and measurements of itch, physical skin response, and subjective skin response were taken. At the end of the session, participants were asked to fill in a final questionnaire assessing the general amount of itch experienced during both baseline and post-VS iontophoresis and, for the open-label groups only, how believable and convincing participants thought the open-label rationale was (on a 0–10 NRS). Upon completion, they were debriefed on the true purpose of the study. For each session, participants received a compensation of €7.50.

### Statistical Analyses

As input for the power calculation, we used the effect size of Cohen’s *d* = 1.10, that was found by Napadow et al. (25), who investigated nocebo effects induced by an inert substance (i.e., a sham allergen solution) on itch. As the current study investigated not only nocebo effects following application of an inert substance but also placebo effects, and both were investigated under closed-label and open-label conditions, a more conservative effect size of *d* = 0.90 was used for computation of sample sizes for the separate open-label and closed-label analyses. A power calculation for an analysis of covariance (ANCOVA) using G*Power 3.1 (45) indicated that 21 participants per group would be needed at a power of β = .80 and a significance level of α = .05 for the primary outcome of AUC itch during iontophoresis in the experimental session between the (separate closed-label or open-label) positive verbal suggestion group and the negative verbal suggestion group while controlling for AUC itch at baseline. A missing data rate of 10% was taken into account, resulting in a sample size of 23 participants in each group.

Analyses were performed using SPSS 21.0 for Windows (IBM SPSS Inc., Chicago, IL, USA). Normal distribution of the variables and the assumptions of each analysis were checked prior to analysis. To test for group differences in demographics and baseline variables, chi-square tests and one-way analyses of variance (ANOVAs) were conducted. As *a priori* determined primary analysis, differences between the combined negative VS groups and positive VS groups in AUC itch during iontophoresis were assessed by a general linear model (GLM) ANCOVA, controlled for AUC itch during baseline iontophoresis. Similar analyses were conducted for the secondary outcome parameters, maximum itch during iontophoresis, mean itch (assessed verbally following iontophoresis), AUC itch during the 4-min follow-up, subjective skin response, and the physical skin response parameters.

Due to technical difficulties with the NRS bar slide and E-Prime, data of some participants (*n* = 6) were missing for the analyses of AUC itch and maximum itch during iontophoresis. Data of one participant were missing for the skin temperature measurements. For those variables that were non-normally distributed (i.e., AUC itch during follow-up), a change score was calculated by subtracting baseline scores from those measured post-VS (with zero indicating no change, negative scores indicating a decrease, and positive scores indicating an increase from baseline to post-VS). A GLM ANOVA was then performed to detect differences in change scores between groups. For expected itch following suggestions, an ANOVA was also conducted. For each AN(C)OVA, Cohen’s *d* was calculated, and the following interpretations were used: small effect size 0.20, medium effect size 0.50, and large effect size 0.80 (46). When appropriate, covariate-adjusted means were used for calculation of Cohen’s *d*. In addition, paired sample *t*-tests were conducted within each group to assess changes in each outcome parameter from the baseline to post-VS measurements. In order to examine whether the effects of verbal suggestions were similar regardless of participants knowing about the expectation induction, all analyses were repeated for the separate open-label and closed-label conditions. As the effects of suggestions were expected to be similar under open-label and closed-label contexts, differences between open- and closed-label groups were not tested statistically. Rather, effect sizes generated by the separate open-label and closed-label analyses were used for indirect comparisons.

To explore potential group differences in the strength of associations between the process measure of post-VS itch expectation and the outcome measures of itch and skin response, Pearson’s *r* correlations were calculated within each group, and Cohen’s *q* was computed as an effect size for the difference in strength of association, with the following categories of interpretation: no effect < 0.10, small effect size 0.10 < 0.30, medium effect size 0.30 < 0.50, and large effect size ≥ 0.50 (46). For AUC itch during follow-up, Spearman’s rho was calculated. The open-label groups were compared on how believable and convincing participants thought the open-label rationale was by independent-samples *t*-tests. All analyses were conducted two sided with α = .05. For the secondary analyses (i.e., AN(C)OVAs and paired-sample *t*-tests for separate open-label and closed-label analyses), Bonferroni’s correction for multiple comparisons was used, thus resulting in a significance level of α/2 = .025. To correct for alpha inflation due to multiple itch outcomes, an additional Bonferroni’s correction was applied for the secondary itch outcomes, resulting in a significance level of α/3 = .017 for the combined-group analyses and (α/3)/2 = .008 for the separate-group analyses of the secondary itch outcomes. All values described in the Results section represent mean ± SD, unless stated otherwise.

## Results

### Participants

A total of 138 potential participants expressed interest in the study, of whom 44 were not included (18 had somatic or psychological morbidity, 4 were non-proficient in the Dutch language, and 22 gave no response following screening). Two participants dropped out after the baseline session and were replaced. This resulted in the intended sample size of 92 participants (16 males, 17.4%; 76 females, 82.6%), whose age ranged from 18 to 30 (*M* = 21.8 ± 2.7). Participants were randomized into 1) the open-label positive VS group (*n* = 22), 2) the closed-label positive VS group (*n* = 23), 3) the open-label negative VS group (*n* = 23), or 4) the closed-label negative VS group (*n* = 24). The groups did not differ in demographic factors (all *p* ≥ .42), baseline itch expectation prior to iontophoresis (*p* *= .*13), baseline self-reported itch parameters (all *p* ≥ .58), and baseline subjective and physical skin condition (all *p* ≥ .12). An overview of the means and standard deviations of the baseline and outcome measures is presented in **Table 1** (combined open-label and closed-label groups) and in **Supplementary Table S1** (separate open-label and closed-label groups).

**Table 1:** Means ± standard deviations for the combined open- and closed-label positive and the combined open- and closed-label negative verbal suggestion groups.

	Combined open- and -closed-label				
	*n*	Positive VS (*n* = 45)	Negative VS (*n* = 47)	AN(C)OVA	
				*p*-value	Cohen’s *d*
*Process measure*					
Pre-iontophoresis itch expectation	92	5.15 ± 1.95	4.82 ± 1.75	.40	
Post-VS itch expectation^A^	92	2.62 ± 1.82	4.41 ± 1.93	<.001	0.96
*Baseline histamine iontophoresis*					
AUC itch^B^	88	369.79 ± 241.69	361.35 ± 230.24	.87	
Maximum itch	88	3.95 ± 2.44	3.78 ± 2.26	.73	
Mean itch^C^	92	3.10 ± 1.90	2.93 ± 1.75	.66	
*Post-VS histamine iontophoresis*					
AUC itch^B,D^	86	314.61 ± 237.34	367.54 ± 266.63	.19	0.29
Maximum itch^D^	86	3.44 ± 2.54	3.81 ± 2.45	.24	0.26
Mean itch^C,D^	92	2.83 ± 1.93	3.19 ± 2.09	.076	0.38
*Change from baseline to post-VS scores*					
AUC itch during follow-up^B,E^	90	−3.38 ± 6.37	0.02 ± 6.88	.017	0.52
*Baseline skin response to iontophoresis*					
Subjective skin response^F^	92	24.37 ± 11.77	22.78 ± 12.25	.53	
Wheal area (cm^2^)	92	10.52 ± 3.47	11.09 ± 3.00	.40	
Flare area (cm^2^)	92	47.74 ± 11.05	48.16 ± 12.45	.86	
Change in skin temperature (°C)^G^	91	1.70 ± 1.01	1.58 ± 1.22	.61	
*Post-VS skin response to iontophoresis*					
Subjective skin response^D,F^	91	21.08 ± 12.31	20.79 ± 12.21	.58	0.12
Wheal area (cm^2^)^D^	92	10.12 ± 3.80	10.68 ± 3.69	.78	0.06
Flare area (cm^2^)^D^	92	45.54 ± 13.11	47.17 ± 11.75	.54	0.13
Change in skin temperature (°C)^D,G^	90	1.83 ± 1.15	2.34 ± 1.62	.018	0.52

^A^VS, verbal suggestions. ^B^AUC, area under the curve. ^C^Assessed verbally on a Numeric Rating Scale ranging from 0 to 10. ^D^Group differences assessed by ANCOVA, controlled for baseline. Cohen’s d was calculated with the estimated marginal means (controlled for baseline). ^E^Calculated as post-VS measure–baseline measure (session 2–session 1) and corrected for significant outliers. ^F^As measured by an adjusted version of the Sensitive Scale-10 (43). ^G^Calculated as post-iontophoresis temperature–pre-iontophoresis temperature.

### Expected Itch

**Figure 2:**
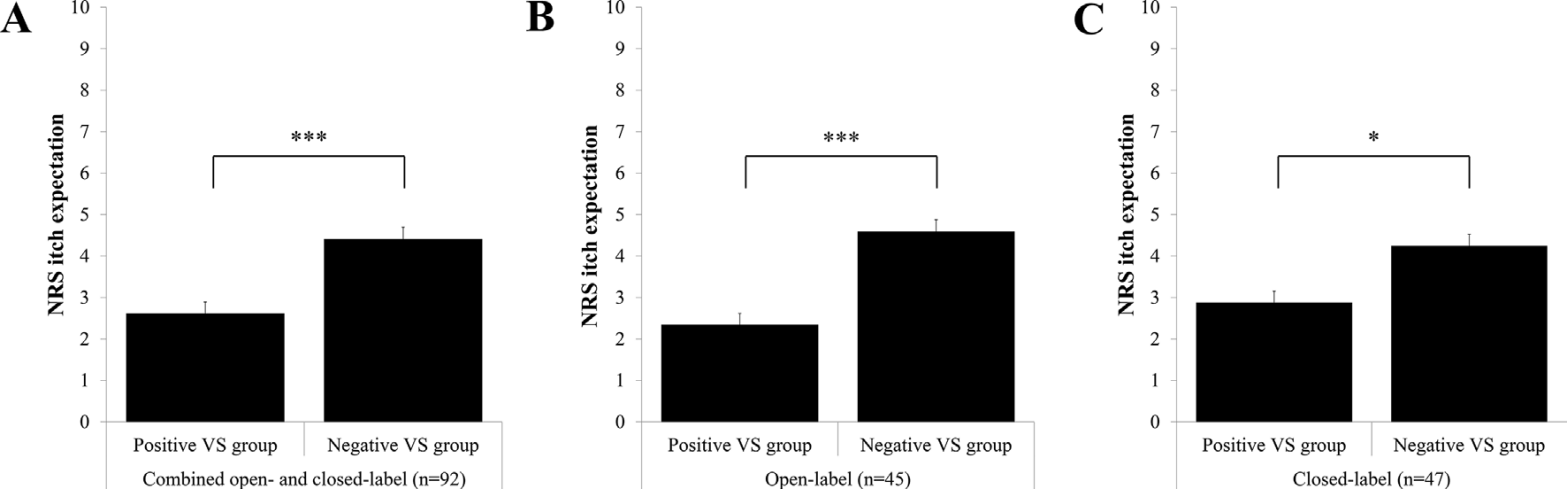
Mean Numeric Rating Scale (NRS) score for post-verbal suggestions (VS) itch expectation with the standard error of the mean (SEM) for **(A)** the combined open- and closed-label positive VS group (*n* = 45) and negative VS group (*n* = 47); **(B)** the open-label positive VS group (*n* = 22) and open-label negative VS group (*n* = 23); and **(C)** the closed-label positive VS group (*n* = 23) and closed-label negative VS group (*n* = 24). ***p < .001, *p < .05.

A large-sized effect of verbal suggestions on expected itch was found; *F*(1,90) = 20.94, *p* < .001, Cohen’s *d* = 0.96. As illustrated in **Figure 2**, expected itch following suggestions was significantly lower in the combined positive VS groups (*M* = 2.62 ± 1.82) compared with the combined negative VS groups (*M* = 4.41 ± 1.93).

A secondary analysis showed a large-sized effect of suggestions in the open-label groups [*F*(1,43) = 15.84, *p* < .001, Cohen’s *d* = 1.21] and a medium-sized effect in the closed-label groups [*F*(1,45) = 6.15, *p* = .017, Cohen’s *d* = 0.74], both indicating significantly lower expected itch in the positive VS group (open label: *M* = 2.35 ± 1.88; closed label: *M* = 2.88 ± 1.77) than in the negative VS group (open label: *M* = 4.59 ± 1.91; closed label: *M* = 4.24 ± 1.99).

### Primary Itch Measure: Area Under the Curve (AUC) of Itch during Histamine Iontophoresis

**Figure 3:**
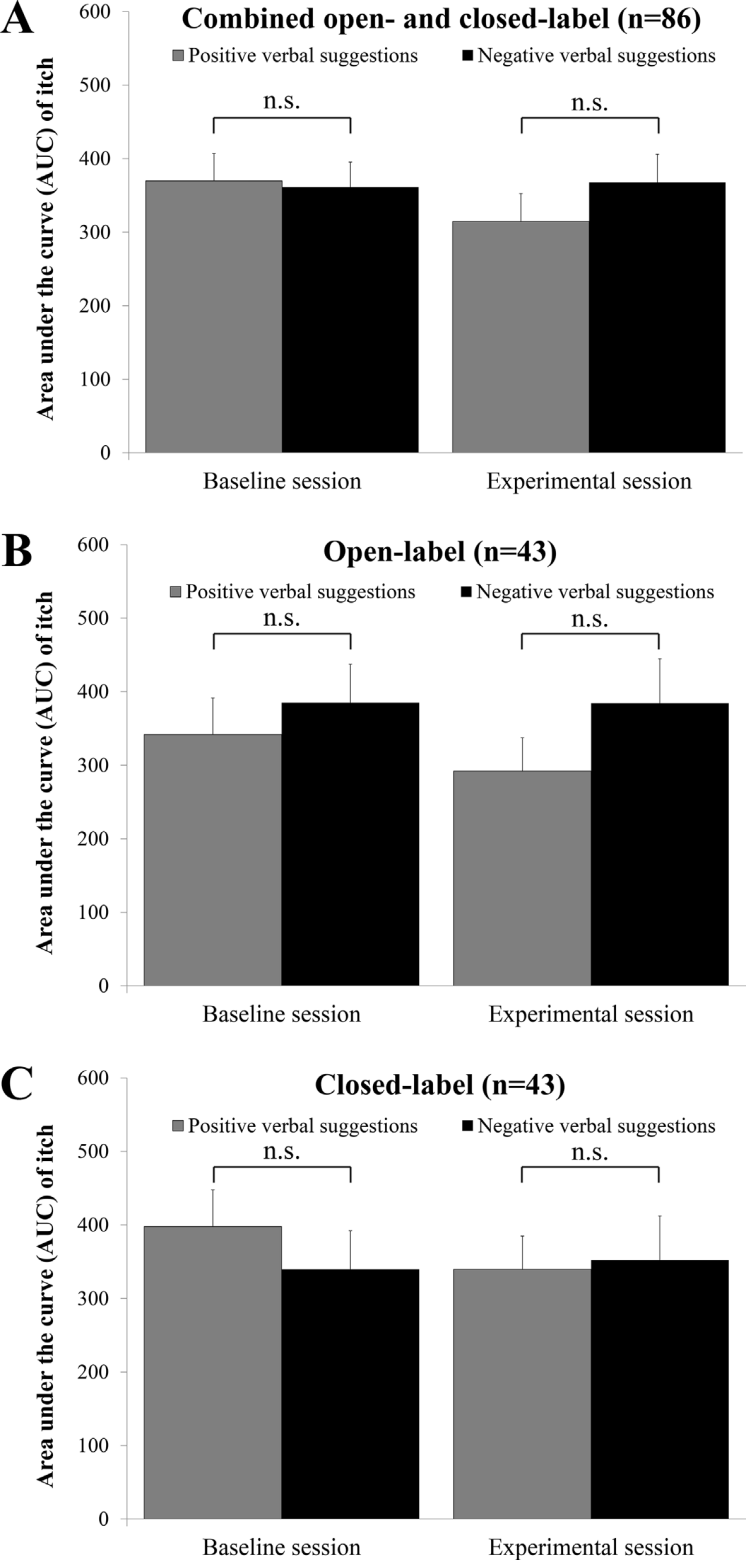
Mean area under the curve (AUC) of self-rated itch during histamine iontophoresis in the baseline and experimental session, with the standard error of the mean (SEM) for **(A)** the combined open- and closed-label positive VS group (*n* = 40) and negative VS group (*n* = 46); **(B)** the open-label positive VS group (*n* = 21) and open-label negative VS group (*n* = 22); and **(C)** the closed-label positive VS group (*n* = 19) and closed-label negative VS group (*n* = 24). n.s. = not significant (*p* > .05).

For the primary outcome AUC itch, a small-sized non-significant difference between the combined positive VS groups and the combined negative VS groups was found; *F*(1,83) = 1.75, *p* = .19, Cohen’s *d* = 0.29. Secondary analyses for the separate open- and closed-label groups revealed similar findings (both *p* ≥ .31; see **Figure 3**). Within-group analyses of baseline to post-VS changes indicated that AUC itch decreased marginally in the combined positive VS groups [*t*(39) = 1.98, *p* = .055] but did not change in the combined negative VS groups [*t*(45) = −0.19, *p* = .85]. No within-group changes in AUC itch from baseline to post-VS were detected for the separate open- and closed-label groups (all *p* ≥ .12). An overview of within-group comparisons for AUC itch and other outcome measures is presented in **Table 2** (combined open-label and closed-label groups) and **Supplementary Table S2** (separate open-label and closed-label groups).

**Table 2:** Within-group mean changes from baseline and separate paired sample *t*-test results for the combined open- and closed-label positive verbal suggestion groups and combined negative verbal suggestion groups.

	Combined open- and closed-label positive VS groups (*n* = 45)				Combined open- and closed-label negative VS groups (*n* = 47)			
	*n*	Mean change	*t*	*p*	*n*	Mean change	*t*	*p*
*Histamine iontophoresis*								
AUC itch^A^	40	−46.91	1.98	.055	46	6.19	−0.19	.85
Maximum itch	40	−0.44	2.00	.053	46	0.02	−0.07	.94
Mean itch^B^	45	−0.26	1.34	.19	47	0.26	−1.30	.20
*Post-iontophoresis follow-up*								
AUC itch^A^	43	−3.73	3.24	.002	47	0.02	−0.02	.98
*Skin response to iontophoresis*								
Subjective skin response^C^	44	−3.30	2.59	.013	47	−2.00	1.61	.12
Wheal area (cm^2^)	45	−0.40	0.79	.43	47	−0.41	0.87	.39
Flare area (cm^2^)	45	−2.20	1.31	.20	47	−0.99	0.50	.62
Change in skin temperature (°C)^D^	44	0.14	−0.88	.38	46	0.76	−3.88	<.001

Mean change was calculated as post-verbal suggestions score–baseline score, with negative values indicating a decrease from baseline and positive scores indicating an increase from baseline. ^A^AUC, area under the curve. ^B^Assessed verbally on a Numeric Rating Scale ranging from 0 to 10. ^C^As measured by an adjusted version of the Sensitive Scale-10 (43). ^D^Calculated as post-iontophoresis temperature–pre-iontophoresis temperature.

### Secondary Itch Measures during and Following Histamine Iontophoresis

#### Maximum Itch and Mean Itch during Iontophoresis

Findings for maximum itch during iontophoresis were similar to those of AUC itch, with no effects of suggestions for the combined as well as separate groups (all *p* ≥ .24) and a marginal decrease from baseline to post-VS exclusively for the combined positive VS groups [*t*(39) = 2.00, *p* = .053]. The combined positive VS groups showed a small-sized tendency to report lower (post-iontophoresis-assessed) mean itch (*M* = 2.83 ± 1.93) than did the combined negative VS groups (*M* = 3.19 ± 2.09); *F*(1,89) = 3.22, *p* = .076, Cohen’s *d* = 0.38. No effects of verbal suggestions were found when open- and closed-label groups were separated, nor were changes from baseline to post-VS scores detected for any of the groups (all *p* ≥ .19).

#### AUC of Itch during Follow-Up Following Iontophoresis

A significant and medium-sized difference in the change scores of AUC for itch during the 4-min follow-up was found when open- and closed-label groups were combined [*F*(1,88) = 6.09, *p* = .016, Cohen’s *d* = 0.52], with AUC itch during follow-up decreasing significantly in the combined positive VS groups (*M* = −3.73 ± 7.55) compared with the combined negative VS groups (*M* = 0.02 ± 6.88). A small-sized non-significant effect of verbal suggestions was found in the open-label groups; *F*(1,43) = 2.11, *p* = .15, Cohen’s *d* = 0.43, and a marginal and medium-sized effect in the closed-label groups, in the same direction as for the combined groups; *F*(1,43) = 4.94, *p* = .032, Cohen’s *d* = 0.67. A significant change from baseline to post-VS in AUC itch during follow-up was demonstrated for the combined positive VS groups [*t*(42) = 3.24, *p* = .002]. In the combined negative VS groups, however, no change was detected [*t*(46) = −0.02, *p* = .98]. Separating open- and closed-label groups revealed a non-significant change within the open-label positive VS group [*t*(21) = 1.87, *p* = .075] and a significant change within the closed-label positive VS group [*t*(20) = 3.14, *p* = .005].

### Skin Response

#### Subjective Skin Response (SS-10)

For subjective skin response following the histamine test, no significant difference was found between the combined positive and negative VS groups, nor between the separate open- and closed-label positive and negative VS groups (all *p* ≥ .12). A significant decrease in subjective skin response from baseline to post-VS was demonstrated in the combined positive VS groups [*t*(43) = 2.59, *p* = .013], but not in the negative VS groups [*t*(46) = 1.61, *p* = .12]. When analyses were conducted for separate open- and closed-label groups, a significant decrease was demonstrated only for the closed-label positive VS group; *t*(22) = 3.75, *p* < .001.

#### Physical Skin Response

No effects of verbal suggestions on wheal or flare areas were found for either the combined or separate open- and closed-label groups (all *p* ≥ .23). Regarding skin temperature, the combined positive VS groups showed a medium-sized lower increase in skin temperature from before to after iontophoresis (*M* = 1.83 ± 1.15) than did the combined negative VS groups (*M* = 2.34 ± 1.62); *F*(1,87) = 5.84, *p* = .018, Cohen’s *d* = 0.52. In the same direction, marginally significant medium-sized effects of verbal suggestions on skin temperature increase were found in the open-label [*F*(1,41) = 3.01, *p* = .090, Cohen’s *d* = 0.54] and closed-label groups [*F*(1,43) = 2.93, *p* = .094, Cohen’s *d* = 0.52], respectively. Within-group comparisons for both combined and separate open- and closed-label positive and negative VS groups showed that skin temperature increased significantly from baseline to post-VS for the negative VS groups (all *p* ≤ .048), but not for the positive VS groups (all *p* ≥ .12).

### Associations between Expected Itch and the Outcome Measures of Itch

In the combined open- and closed-label groups, expected itch following suggestions was significantly and positively associated with all itch measures during and following iontophoresis (all *r* ≥ .43, all *p* ≤ .01). Comparisons of the strength of the association between expected itch and the itch outcome measures showed small-sized to no differences in associative strength between the combined positive and combined negative VS groups (all Cohen’s *q* ≤ 0.15). In the separate open-label and closed-label groups, findings were similar, with one exception: in the open-label positive VS group exclusively, itch expectations were not associated with mean itch and AUC of itch during follow-up (both *p* ≥ .11). An overview of Pearson’s *r* and Spearman’s ρ correlation coefficients can be found in **Table 3** (combined open- and closed-label groups) and **Supplementary Table S3** (separate open- and closed-label groups).

**Table 3:** Within-group Pearson’s *r* and Spearman’s rho correlations for the process measure of post-VS itch expectation and outcome measures of self-reported itch and skin response for the combined open- and closed-label group comparisons, with Cohen’s *q* as estimate of the difference in effect size between groups.

	Combined open- and closed-label groups		
	Positive VS (*n* = 45)	Negative VS (*n* = 47)	Cohen’s *q*
*Post-VS histamine iontophoresis*			
AUC itch^A^	.67***	.58***	0.15
Maximum itch	.63***	.59***	0.06
Mean itch^B^	.52***	.60***	0.12
*Post-VS follow-up on iontophoresis*			
AUC itch during follow-up^A,C^	.43**	.49***	0.08
*Post-VS skin response to iontophoresis*			
Subjective skin response^D^	.50***	.59***	0.13
Wheal area (cm^2^)	−.09	−.01	0.08
Flare area (cm^2^)	.03	−.23	0.26
Change in skin temperature (°C)^E^	.04	−.19	0.23

^A^AUC, area under the curve. ^B^Assessed verbally on a Numeric Rating Scale ranging from 0 to 10. ^C^Calculated using the non-parametric Spearman’s rho. ^D^As measured by an adjusted version of the Sensitive Scale-10 (43). ^E^Calculated as post-iontophoresis temperature–pre-iontophoresis temperature. **p < .01; ***p < .001.

### Open-Label Instruction Believability

Overall, participants in the open-label conditions rated the instructions as very clear (*M* = 7.90 ± 2.32). Ratings on how convincing the instructions had been were more ambiguous (*M* = 5.37 ± 2.46). In general, participants in the open-label groups believed that expectations are able to influence itch (*M* = 6.49 ± 1.97) but rated the extent in which their own itch experience was influenced by the application of the tonic as low (*M* = 3.81 ± 2.43). Groups did not differ in their ratings of the instructions (all *p* ≥ .21).

## Discussion

The current study investigated whether positive and negative outcome expectations, induced by open-label and closed-label positive and negative verbal suggestions regarding an inert tonic, could influence self-reported itch in response to a histamine test. For the first time, open- and closed-label placebo effects for itch were investigated within a single study, including a comparison with open- and closed-label nocebo effects. It was demonstrated that both open-label and closed-label verbal suggestions were able to influence itch expectations. For the primary outcome of area under the curve for itch during histamine iontophoresis, a small-sized but non-significant effect of verbal suggestions was found. Participants in the combined open- and closed-label positive VS groups reported lower itch during an immediate follow-up period after iontophoresis compared to the negative VS groups. *Post hoc* tests indicated that this was mostly due to differences between positive and negative VS groups under closed-label conditions. In addition, a significantly smaller increase in skin temperature was observed in the combined positive VS groups compared with the negative VS groups, but no effects on other markers of the physical skin response to histamine were found. Overall, the current study shows that verbal suggestions regarding a topical application of a substance can influence expectations for itch, regardless of whether or not participants know about receiving suggestions, and provides limited evidence that these suggestions may influence itch and skin response in response to histamine.

The findings that verbal suggestions were able to influence itch in the follow-up period after histamine iontophoresis are in line with a previous study that found medium-to-large-sized effects of positive suggestions on histamine-induced itch (24). While that particular study made use of a cream to help induce placebo effects, the current study used a pink-colored tonic. Potentially, the use of this particular attribute may have led towards smaller effects in the current study, since a cream could be perceived as a common treatment for itch by some participants, could trigger previously learned associations, and could thus potentially elicit stronger effects overall (47). Moreover, negative verbal suggestions did not elicit negative expectations for itch in the current study and did not increase itch either during or following the histamine test, which is not in line with previous evidence for verbal suggestion-induced nocebo effects in itch (25, 26, 28). It should be noted though that these previous studies have induced nocebo effects through negative suggestions regarding the experimental itch induction method that was used, whereas the current study provided suggestions regarding the topical application of an attribute prior to itch induction. While this did allow for a direct comparison of positive and negative expectation induction, potentially, it may have influenced the credibility of the negative verbal suggestions as well. Topical application of, for example, a cream or tonic in a laboratory setting might be associated more easily with symptom reduction rather than worsening of symptoms. In comparison, information regarding an experimental itch induction method, though less clinically relevant, may provide a more neutral basis for induction of nocebo effects through suggestions. Alternatively, although the baseline histamine application was valuable for participants as a comparison point for the second application, nocebo effects induced through negative verbal suggestions could have been influenced by participants being less anxious about the second histamine test, in comparison with the first test (since participants were generally unfamiliar with histamine iontophoresis prior to participating in the study). Future research may utilize a counterbalanced design to examine this more in detail. Likewise, more research is needed to investigate under which circumstances and through which attributes placebo and nocebo effects may be elicited for itch.

An effect of negative verbal suggestions on change in skin temperature due to histamine application was demonstrated. This finding is similar to previous work on placebo effects in autonomically controlled parameters and wheal responses (26, 48), a meta-analysis of clinical trials demonstrating placebo effects on physical outcome parameters controlled by the autonomic nervous system (49), and early studies on suggestions and hypnosis (50–52). Considering that either the outcome measure differed from these previous studies (i.e., skin temperature change rather than wheal size) or the expectation induction method was different (i.e., verbal suggestions given without hypnosis), caution is needed in interpreting these results. Moreover, the verbal suggestions in the current study did not influence wheal and flare areas to histamine, which is in line with most recent studies (24, 29, 35, 53, 54).

Our design allowed for the first time comparisons of effect sizes of positive and negative verbal suggestions under open- and closed-label conditions for itch. The findings demonstrate that positive verbal suggestions are able to significantly reduce expectations of itch under both open-label and closed-label conditions, with open-label verbal suggestions seemingly inducing larger expectancy effects. Overall, the effects of positive and negative verbal suggestions on itch were approximately similar sized under open-label and closed-label conditions. However, some differences between the conditions could be seen when examining the within-group changes from baseline. Closed-label suggestions appeared slightly more effective for itch, as illustrated by the significant within-group changes in itch during follow-up from baseline to post-suggestions under closed-label conditions. That open-label placebo treatment can significantly influence expectations and, potentially, symptoms of itch is in line with previous findings on other outcome parameters (31, 32, 34–39). It also provides further preliminary support for the notion that concealment of treatment is not necessary to elicit placebo responses, and that placebo mechanisms can potentially be utilized in clinical practice. Small differences between the open-label instructions of the current study and previous work need to be noted. Previous studies [e.g., Refs. 31–34, 40] began their open-label placebo instructions by indicating that the pill that was used was a placebo, prior to indicating the efficacy and mechanisms of these effects. The current study on the other hand began by introducing the tonic as an effective tool for itch reduction and explaining that it was a placebo afterwards, together with a rationale on why it would still be effective. Differences in the order in which this type of information is presented may impact the strength of open-label placebo and nocebo effects. In addition, previous work has incorporated the concept of learning in the open-label instructions (i.e., by giving the example of Pavlov’s dog). This aspect has been omitted here, as the current study investigates placebo responses evoked by conscious expectancy (i.e., verbal information) rather than associative learning mechanisms. Potentially, this may have influenced the efficacy of the open-label rationale. Some caution needs to be taken in interpreting the effects of negative verbal suggestions under the separate open-label and closed-label conditions, since neither type of negative verbal suggestions was able to increase expectations of itch.

Some strengths and limitations need to be taken into account. This is the first study that compares open- and closed-label positive and negative verbal suggestions to elicit placebo and nocebo effects in itch and other responses to histamine. Since the study was conducted single blinded, a reporting bias cannot be ruled out, as participants may have adjusted their answers to the experimenters’ expectations. To minimize influences of response bias on assessments of expectations and itch, participants used a (computerized) bar slide to indicate these parameters. Future research might, however, consider using a double-blinded approach. The effect sizes found in the current study are considerably small, which may be due to the itch stimulus being perceived as low by participants. As such, the study may have been underpowered to find small effects, which seems to be supported by finding more significant effects of the combined open- and closed-label groups than for the separate groups. Moreover, the design of the current study did not include a no-treatment group. This prevents an estimation of a true placebo or nocebo response, as itch may reduce from the first to second histamine test regardless of group allocation. Though habituation to the itch stimulus cannot be ruled out, its role is likely small, since the itch stimuli were relatively short and presented with 1 week in between. Alternatively, anxiety may have resulted in higher itch ratings during baseline. Including a no-treatment group to control for these reductions or utilizing a counterbalanced design could provide better estimates of a true placebo and nocebo response. Lastly, verbal suggestions were given regarding an inert tonic. While this approach may have worked for placebo induction, potentially, it may have been harder to elicit nocebo effects in this manner, as negative consequences regarding such a treatment method may be counterintuitive. To compare open-label and closed-label nocebo effects for itch, a different approach could be needed. For example, future research could investigate whether nocebo effects can be induced when the effects of an inert substance on itch are introduced as side effects of this substance, as changing to such an introduction of negative effects may be more closely related to how negative effects would be experienced in clinical practice.

In conclusion, this study provides evidence for the first time that positive verbal suggestions can induce expectations for itch reduction under both open-label and closed-label conditions. Suggestions are able to reduce the amount of itch experienced after histamine iontophoresis under both open-label and closed-label conditions, with closed-label suggestions appearing more effective in reducing itch during follow-up. However, experienced itch during histamine iontophoresis was not influenced by suggestions. Future research may aim to investigate under which circumstances and with which type of attribute these suggestions could elicit effects for itch. Further demonstrating the efficacy of open-label placebo effects may help facilitate the application of these effects in clinical practice.

## Author Contributions

SM, HvM, and AE designed the study and wrote the protocol. AvL and DV commented on the protocol. SM and AvL undertook the statistical analysis. SM and HvM wrote the first draft of the manuscript. AvL, DV, AL and AE commented on the manuscript.

## Funding

This study was funded by a European Research Council Consolidator Grant 2013 (ID: ERC-2013-CoG-617700_EXPECT HEAL-TH, granted to AE). The funders had no role in study design, data collection or analysis, decision to publish, or writing this manuscript.

## Conflict of Interest Statement

The authors declare that the research was conducted in the absence of any commercial or financial relationships that could be construed as a potential conflict of interest.
