# Supplementary material for: Effects of Open- and Closed-Label Nocebo and Placebo Suggestions on Itch and Itch Expectations
Source: Front Psychiatry. 2019 Jun 21;10:436. doi: 10.3389/fpsyt.2019.00436 (PMC6598628; doi:10.3389/fpsyt.2019.00436)
Supplement: Supplementary file 1 [file DataSheet_1.docx]

Supplementary Material

Effects of open- and closed-label nocebo and placebo suggestions on itch and itch expectancies

Stefanie H. Meeuwis^1,2*^, Henriët van Middendorp^1,2^, Antoinette I.M. van Laarhoven^1,2,4^, Dieuwke S. Veldhuijzen^1,2^, Adriana P.M. Lavrijsen^3^, Andrea W.M. Evers^1,2,4^

^1^ Leiden University, Faculty of Social and Behavioural Sciences, Institute of Psychology, Health, Medical and Neuropsychology Unit, Leiden, The Netherlands

^2^ Leiden Institute for Brain and Cognition, Leiden University Medical Center, Leiden, The Netherlands

^3^ Department of Dermatology, Leiden University Medical Center, Leiden, The Netherlands

^4^ Department of Psychiatry, Leiden University Medical Center, Leiden, The Netherlands

*** Correspondence:**

Stefanie H. Meeuwis,

s.h.meeuwis@fsw.leidenuniv.nl

**Supplementary Table S1.** Means ± standard deviations for the separate open- and closed-label positive and the negative verbal suggestion groups.

|  |  | | | | |  |  | | | | |
| --- | --- | --- | --- | --- | --- | --- | --- | --- | --- | --- | --- |
|  | **Open-label groups** | | | | |  | **Closed-label groups** | | | | |
|  |  | | | **AN(C)OVA** | |  |  | | | **AN(C)OVA** | |
|  | ***n*** | **Positive VS (*n*=22)** | **Negative VS (*n*=23)** | ***p*-value** | **Cohen’s *d*** |  | ***n*** | **Positive VS (*n*=23)** | **Negative VS (*n*=24)** | ***p*-value** | **Cohen’s *d*** |
|  |  |  |  |  |  |  |  |  |  |  |  |
| *Process measure* |  |  |  |  |  |  |  |  |  |  |  |
| Pre-iontophoresis itch expectation | 45 | 4.87 ± 1.93 | 5.37 ± 1.40 | .32 |  |  | 47 | 5.41 ± 1.97 | 4.29 ± 1.92 | .055 |  |
| Post-VS itch expectation ^A^ | 45 | 2.35 ± 1.88 | 4.59 ± 1.91 | <.001 | 1.21 |  | 47 | 2.88 ± 1.77 | 4.24 ± 1.99 | .017 | 0.74 |
|  |  |  |  |  |  |  |  |  |  |  |  |
| *Baseline histamine iontophoresis* |  |  |  |  |  |  |  |  |  |  |  |
| AUC itch ^B^ | 43 | 341.78 ± 228.13 | 384.96 ± 245.59 | .55 |  |  | 45 | 397.79 ± 257.03 | 339.70 ± 218.22 | .42 |  |
| Maximum itch | 43 | 3.75 ± 2.47 | 4.00 ± 2.31 | .73 |  |  | 45 | 4.16 ± 2.45 | 3.58 ± 2.24 | .42 |  |
| Mean itch ^C^ | 45 | 2.84 ± 1.77 | 3.11 ± 1.89 | .62 |  |  | 47 | 3.34 ± 2.03 | 2.75 ± 1.63 | .27 |  |
|  |  |  |  |  |  |  |  |  |  |  |  |
| *Post-VS histamine iontophoresis* |  |  |  |  |  |  |  |  |  |  |  |
| AUC itch ^B,D^ | 43 | 292.07 ± 217.57 | 384.44 ± 296.16 | .31 | 0.32 |  | 43 | 339.52 ± 261.15 | 352.05 ± 241.83 | .47 | 0.23 |
| Maximum itch ^D^ | 43 | 3.27 ± 2.65 | 3.91 ± 2.70 | .45 | 0.24 |  | 43 | 3.64 ± 2.47 | 3.71 ± 2.24 | .42 | 0.26 |
| Mean itch ^C,D^ | 45 | 2.59 ± 1.70 | 3.29 ± 2.11 | .20 | 0.40 |  | 47 | 3.06 ± 2.14 | 3.10 ± 2.12 | .22 | 0.37 |
|  |  |  |  |  |  |  |  |  |  |  |  |
| *Change from baseline to post-VS scores* |  |  |  |  |  |  |  |  |  |  |  |
| AUC itch during follow-up ^B,E^ | 45 | -2.98 ± 7.19 | 0.05 ± 7.97 | .19 | 0.41 |  | 45 | -3.79 ± 5.54 | -0.01 ± 5.83 | .032 | 0.68 |
|  |  |  |  |  |  |  |  |  |  |  |  |
| *Baseline skin response to iontophoresis* |  |  |  |  |  |  |  |  |  |  |  |
| Subjective skin response ^F^ | 44 | 24.65 ± 11.17 | 24.31 ± 12.98 | .93 |  |  | 47 | 24.12 ± 12.53 | 21.32 ± 11.59 | .43 |  |
| Wheal area [cm^2^] | 45 | 11.30 ± 3.01 | 11.31 ± 3.17 | .997 |  |  | 47 | 9.76 ± 3.78 | 10.89 ± 2.89 | .26 |  |
| Flare area [cm^2^] | 45 | 47.02 ± 11.34 | 50.94 ± 11.53 | .26 |  |  | 47 | 48.44 ± 10.97 | 45.51 ± 12.96 | .41 |  |
| Change in skin temperature [°C] ^G^ | 45 | 1.57 ± 1.03 | 1.60 ± 0.96 | .93 |  |  | 46 | 1.83 ± 1.00 | 1.57 ± 1.46 | .48 |  |
|  |  |  |  |  |  |  |  |  |  |  |  |
| *Post-VS skin response to iontophoresis* |  |  |  |  |  |  |  |  |  |  |  |
| Subjective skin response ^D,F^ | 44 | 23.60 ± 14.19 | 21.33 ± 11.54 | .43 | 0.24 |  | 47 | 18.77 ± 10.07 | 20.27 ± 13.06 | .12 | 0.47 |
| Wheal area [cm^2^] ^D^ | 45 | 10.17 ± 3.40 | 11.27 ± 3.04 | .23 | 0.37 |  | 47 | 10.07 ± 4.22 | 10.11 ± 4.22 | .32 | 0.30 |
| Flare area [cm^2^] ^E.H^ | 45 | 43.00 ± 10.88 | 50.93 ± 11.88 | .054 | 0.33 |  | 47 | 47.97 ± 14.76 | 43.58 ± 10.66 | .25 | 0.12 |
| Change in skin temperature [°C] ^D,G^ | 44 | 1.91 ± 0.88 | 2.51 ± 1.50 | .090 | 0.54 |  | 46 | 1.76 ± 1.36 | 2.16 ± 1.74 | .094 | 0.52 |
|  |  |  |  |  |  |  |  |  |  |  |  |

Note. ^A^ VS = verbal suggestions. ^B^ AUC = Area under the Curve. ^C^ Assessed verbally on a Numeric Rating Scale ranging from 0-10. ^D^ Group differences assessed by ANCOVA, controlled for baseline. Cohen’s d was calculated with the estimated marginal means (controlled for baseline). ^E^ Calculated as post-VS measure – baseline measure (session 2 – session 1) and corrected for significant outliers. ^F^ As measured by an adjusted version of the Sensitive Scale 10 (Misery et al., 2014). ^G^ Calculated as post-iontophoresis temperature – pre-iontophoresis temperature. ^H^ For flare area, an ANOVA was conducted as homogeneity of regression slopes was unequal.

**Supplementary Table S2.** Within-group mean changes from baseline and separate paired sample t-test results for the open- and closed-label positive verbal suggestion groups and negative verbal suggestion groups.

|  |  | | | | | | | | |  |  | | | | | | | | |
| --- | --- | --- | --- | --- | --- | --- | --- | --- | --- | --- | --- | --- | --- | --- | --- | --- | --- | --- | --- |
|  | **Open-label groups** | | | | | | | | |  | **Closed-label groups** | | | | | | | | |
|  | **Positive VS (*n*=22)** | | | |  | **Negative VS (*n*=23)** | | | |  | **Positive VS (*n*=23)** | | | |  | **Negative VS (*n*=24)** | | | |
|  | *n* | Mean change | *t* | *p* |  | *n* | Mean change | *t* | *p* |  | *n* | Mean change | *t* | *p* |  | *n* | Mean change | *t* | *p* |
|  |  |  |  |  |  |  |  |  |  |  |  |  |  |  |  |  |  |  |  |
| *Histamine iontophoresis* |  |  |  |  |  |  |  |  |  |  |  |  |  |  |  |  |  |  |  |
| AUC itch ^A^ | 21 | -49.71 | 1.61 | .12 |  | 22 | -0.52 | 0.01 | .99 |  | 19 | -43.81 | 1.17 | .26 |  | 24 | 12.35 | -0.26 | .80 |
| Maximum itch | 21 | -0.48 | 1.52 | .15 |  | 22 | -0.09 | 0.20 | .84 |  | 19 | -0.39 | 1.26 | .22 |  | 24 | 0.13 | -0.33 | .75 |
| Mean itch ^B^ | 22 | -0.25 | 0.87 | .39 |  | 23 | 0.18 | -0.71 | .48 |  | 23 | -0.28 | 1.00 | .33 |  | 24 | 0.35 | -1.07 | .30 |
|  |  |  |  |  |  |  |  |  |  |  |  |  |  |  |  |  |  |  |  |
| *Post-iontophoresis follow-up* |  |  |  |  |  |  |  |  |  |  |  |  |  |  |  |  |  |  |  |
| AUC itch ^A^ | 22 | -3.68 | 1.87 | .075 |  | 23 | 0.05 | -0.03 | .98 |  | 21 | -3.79 | 3.14 | .005 |  | 24 | -0.01 | 0.01 | .99 |
|  |  |  |  |  |  |  |  |  |  |  |  |  |  |  |  |  |  |  |  |
| *Skin response to iontophoresis* |  |  |  |  |  |  |  |  |  |  |  |  |  |  |  |  |  |  |  |
| Subjective skin response ^C^ | 21 | -1.05 | 0.50 | .62 |  | 23 | -2.98 | 1.91 | .07 |  | 23 | -5.35 | 3.75 | .001 |  | 24 | -1.05 | 0.55 | .59 |
| Wheal area [cm^2^] | 22 | -1.13 | 1.60 | .13 |  | 23 | -0.04 | 0.05 | .96 |  | 23 | 0.31 | -0.45 | .66 |  | 24 | -0.77 | 1.31 | .20 |
| Flare area [cm^2^] | 22 | -4.01 | 1.72 | .10 |  | 23 | -0.01 | <0.01 | >.99 |  | 23 | -0.46 | 0.19 | .85 |  | 24 | -1.93 | 0.68 | .50 |
| Change in skin temperature [°C] ^D^ | 21 | 0.36 | -1.65 | .12 |  | 23 | 0.92 | -3.42 | .002 |  | 23 | -0.07 | 0.30 | .77 |  | 23 | 0.60 | -2.09 | .048 |
|  |  |  |  |  |  |  |  |  |  |  |  |  |  |  |  |  |  |  |  |

Note. Mean change was calculated as post-verbal suggestions score – baseline score, with negative values indicating a decrease from baseline, and positive scores indicating an increase from baseline. ^A^ AUC = Area under the Curve. ^B^ Assessed verbally on a Numeric Rating Scale ranging from 0-10. ^C^ As measured by an adjusted version of the Sensitive Scale 10 (Misery et al., 2014). ^D^ Calculated as post-iontophoresis temperature – pre-iontophoresis temperature.

**Supplementary Table S3.** Within-group Pearson’s r and Spearman’s rho correlations for the process measure of post-VS itch expectation and outcome measures of self-reported itch and skin response for the separate open- and closed-label group comparisons separately.

|  |  | |  |  |  | |  |
| --- | --- | --- | --- | --- | --- | --- | --- |
|  | **Open-label groups** | |  |  | **Closed-label groups** | |  |
|  | **Positive VS (*n*=22)** | **Negative VS (*n*=23)** | **Cohen’s *q*** |  | **Positive VS (*n*=23)** | **Negative VS (*n*=24)** | **Cohen’s *q*** |
| *Post-VS histamine iontophoresis* |  |  |  |  |  |  |  |
| AUC itch ^A^ | .68 *** | .59 ** | 0.15 |  | .67 *** | .57 ** | 0.16 |
| Maximum itch | .56 ** | .59 ** | 0.05 |  | .68 *** | .59 ** | 0.15 |
| Mean itch ^B^ | .33 | .68 *** | 0.49 |  | .68 *** | .53 ** | 0.24 |
|  |  |  |  |  |  |  |  |
| *Post-VS follow-up on iontophoresis* |  |  |  |  |  |  |  |
| AUC itch during follow-up ^A, C^ | .35 | .53 ** | 0.23 |  | .57 ** | .50 * | 0.10 |
|  |  |  |  |  |  |  |  |
| *Post-VS skin response to iontophoresis* |  |  |  |  |  |  |  |
| Subjective skin response ^D^ | .51 * | .52 * | 0.01 |  | .60 ** | .64 *** | 0.07 |
| Wheal area [cm^2^] | -.27 | .12 | 0.40 |  | .06 | -.12 | 0.18 |
| Flare area [cm^2^] | -.21 | -.40 † | 0.21 |  | .17 | -.15 | 0.32 |
| Change in skin temperature [°C] ^E^ | -.43 † | -.32 | 0.13 |  | .35 | -.12 | 0.49 |
|  |  |  |  |  |  |  |  |

Note. ^A^ AUC = Area under the Curve. ^B^ Assessed verbally on a Numeric Rating Scale ranging from 0-10. ^C^ Calculated using the non-parametric Spearman’s rho. ^D^ As measured by an adjusted version of the Sensitive Scale 10 (Misery et al., 2014). ^E^ Calculated as post-iontophoresis temperature – pre-iontophoresis temperature. † p<.10; * p<.05; ** p<.01; *** p<.001
